# Complete Genome Sequences of Two Methicillin-Resistant Staphylococcus haemolyticus Isolates of Multilocus Sequence Type 25, First Detected by Shotgun Metagenomics

**DOI:** 10.1128/genomeA.00036-18

**Published:** 2018-04-05

**Authors:** Natacha Couto, Monika A. Chlebowicz, Erwin C. Raangs, Alex W. Friedrich, John W. Rossen

**Affiliations:** a University of Groningen, University Medical Center Groningen, Department of Medical Microbiology, Groningen, Netherlands

## Abstract

The emergence of nosocomial infections by multidrug-resistant Staphylococcus haemolyticus isolates has been reported in several European countries. Here, we report the first two complete genome sequences of S. haemolyticus sequence type 25 (ST25) isolates 83131A and 83131B. Both isolates were isolated from the same clinical sample and were first identified through shotgun metagenomics.

## GENOME ANNOUNCEMENT

A recent worldwide increase in the number of multidrug-resistant (MDR) Staphylococcus haemolyticus isolates causing hospital infections has been associated with an unusual genome plasticity generated by a large number of insertion sequences (1). In particular, isolates belonging to multilocus sequence type 25 (ST25), initially detected in India (2), are known to cause keratitis and belong to clonal complex 1 (CC1). So far, ST25 isolates have been isolated in several European countries (3).

Here, we report the complete genome sequences of two coexisting ST25 S. haemolyticus isolates from the same clinical sample (peritoneal fluid), originally identified through shotgun metagenomics (N. Coutu, L. Schuele, E.C. Raangs, et al., submitted for publication).

DNA extraction was performed with the DNeasy UltraClean microbial kit (Qiagen, Hilden, Germany). DNA concentration was measured with a Qubit 2.0 fluorometer (Life Technologies, Thermo Fisher Scientific, Waltham, MA). Both strains were sequenced using the MiSeq platform (Illumina, San Diego, CA), generating 2 × 250-bp reads, and the MinION instrument (Oxford Nanopore, Oxford, UK) in order to close the first genomes of this lineage. The Illumina and MinION reads were assembled using Unicycler v0.4.1 (4). Genome annotation was performed at the GenBank submission step using the Prokaryotic Genome Automatic Annotation Pipeline (PGAAP) from NCBI (5).

The genome of the ST25 S. haemolyticus 83131A isolate has a circular chromosome with 2,593,983 bp and a GC content of 32.8%, 2,449 coding sequences (CDS), 60 tRNAs, and 19 rRNAs. The genome of the ST25 S. haemolyticus 83131B isolate has a circular chromosome with 2,593,418 bp and a GC content of 32.8%, 2,437 CDS, 60 tRNAs, and 19 rRNAs. The 83131A isolate harbors one circular plasmid of 16,882 bp (p83131A-A), and the 83131B strain harbors two circular plasmids of 16,882 bp (p83131A-B) and 2,366 bp (p83131B).

The hybrid assembly yielded a contig with the entire genome, which was different from that obtained by using Illumina alone. This is due to the resulting short-read length, which cannot span across repetitive regions, such as transposons and insertion sequences, and which creates gaps in assemblies. Thus, our results reflect the suitability of MinION plus Illumina sequencing for full characterization of plasmids and other mobile genetic elements, which are chimeric elements that are impossible to elucidate by using short-read sequencing approaches alone (5). Through whole-genome MLST (wgMLST) analysis using Ridom SeqSphere+ v4.1.9, we were able to determine that the two isolates differed in 7 out of 2,321 alleles. In the NCBI database, there were only three closed full-genome sequences of S. haemolyticus available. The most closely related genome was from an S. haemolyticus ST42 strain (GenBank accession number CP011116) isolated during a nosocomial outbreak in Brazil (6), which differed in 514 alleles. The observed high genomic diversity among S. haemolyticus genomes shows the need for more full-genome sequences. Genomic comparisons often provide information about similarities and differences of isolates of the same species that belong to different lineages and therefore help identify factors that contribute to the emergence of successful clones.

### Accession number(s).

The GenBank genome accession numbers for the sequences described in this study are CP024809 and CP025396 (genome sequences for isolates 83131A and 83131B, respectively), CP024810 (p83131A-A), CP025397 (p83131A-B), and CP025398 (p83131B).
